# Data-driven magnetohydrodynamic modelling of a flux-emerging active region leading to solar eruption

**DOI:** 10.1038/ncomms11522

**Published:** 2016-05-16

**Authors:** Chaowei Jiang, S. T. Wu, Xuesheng Feng, Qiang Hu

**Affiliations:** 1 SIGMA Weather Group, State Key Laboratory for Space Weather, National Space Science Center, Chinese Academy of Sciences, No.1 Nan-Er-Tiao, Zhong-Guan-Cun, Hai-Dian District, Beijing 100190, China; 2 Center for Space Plasma & Aeronomic Research, The University of Alabama in Huntsville, Huntsville, Alabama 35899, USA

## Abstract

Solar eruptions are well-recognized as major drivers of space weather but what causes them remains an open question. Here we show how an eruption is initiated in a non-potential magnetic flux-emerging region using magnetohydrodynamic modelling driven directly by solar magnetograms. Our model simulates the coronal magnetic field following a long-duration quasi-static evolution to its fast eruption. The field morphology resembles a set of extreme ultraviolet images for the whole process. Study of the magnetic field suggests that in this event, the key transition from the pre-eruptive to eruptive state is due to the establishment of a positive feedback between the upward expansion of internal stressed magnetic arcades of new emergence and an external magnetic reconnection which triggers the eruption. Such a nearly realistic simulation of a solar eruption from origin to onset can provide important insight into its cause, and also has the potential for improving space weather modelling.

## Abstract (web summary)

Solar eruptions heavily influence space weather, but what are their causes remains an open question. Here the authors present a magnetohydrodynamic model of a solar eruption directly driven by solar magnetograms, following the transition from pre-eruptive to eruptive state.

## Graphical abstract

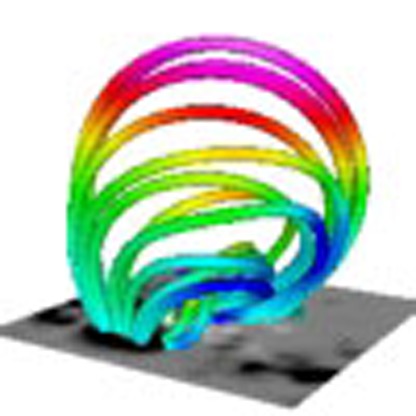

Although manifested diversely as flares, eruptive prominences and coronal mass ejections (CMEs), solar eruptions are essentially explosive release of excess magnetic energy of the Sun's corona. Observations show that solar eruptions can occur abruptly after a quasi-static evolution phase of a few hours to even days during which the magnetic free energy is accumulated^1^,^2^,^3^. There has been an intense debate for decades about what causes such catastrophic disruption of the coronal magnetic field. It is not only a fundamental question in astrophysics, but also has unique importance for space weather, in which solar eruptions play a significant role. Over the past 40 years, a variety of models have been proposed to explain the initiation mechanism of solar eruptions^4^,^5^,^6^,^7^,^8^. Some researchers^9^,^10^ emphasize the importance of ideal magnetohydrodynamic (MHD) instabilities^11^, in particular, the unstable nature of the pre-existing magnetic flux rope^12^,^13^,^14^, which is a volumetric channel of electric current emerging from the convection zone^15^,^16^,^17^ or formed *in situ* in the corona^18^. Others^19^,^20^,^21^ stress the primary role of magnetic reconnection^22^, and believe that without reconnection the eruptions can never happen even if the magnetic energy is excessively supplied. The theoretical models complemented with numerical MHD simulations^9^,^23^,^24^,^25^,^26^,^27^ have greatly improved our understanding of those most violent space weather drivers. All of these models are, however, idealized or hypothetical simplification of the realistic case that is much more complex and elusive in observation.

Existing models that attempt to characterize the realistic magnetic environment for studying solar eruptions are mostly restricted to static reconstruction of the near force-free coronal magnetic field^28^. In this category, the mechanism of eruption can only be investigated tentatively because no dynamics is included. Even a time-sequence of magnetic fields reconstructed following the coronal evolution does not reflect its intrinsic dynamics because these magnetic fields are treated as being independent of each other. There are models^29^,^30^,^31^,^32^ using the reconstructed coronal field immediately preceding eruption (thus the unstable nature of the field has already well-developed) as the initial condition for MHD simulation, which prove to be able to reproduce the fast dynamic phase of the erupting field^30^. However, these kinds of simulations do not self-consistently show how the pre-eruptive field is formed and how the eruption is triggered. Thus such models may not identify the true trigger mechanism.

Here we present a self-consistent MHD simulation of the whole process from the formation to initiation of a coronal eruptive field in a complex multi-polar active region (AR). The event is characterized by a fast magnetic flux emergence of over 2 days leading to an M-class eruptive flare on the 3rd day. Distinct from the aforementioned works, we use a unified MHD model and start it from a very stable state when the coronal field is still near potential (that is, current-free). A 3-day sequential data of surface vector magnetograms are used to drive the coronal magnetic field evolution all the way from its initial potential state to eruption. It is found that the modelled magnetic field evolves stably in the non-eruptive duration of 2 days and becomes unstable at a time instant in good agreement with that of the observed flare eruption. Moreover, the continuously evolving coronal field presents good morphological similarities with the extreme ultraviolet (EUV) emissions. From the simulated magnetic field, we further identify the important role played by magnetic topology changes and magnetic reconnection in leading to the eruption. Detailed analyses are to be presented in the following sections.

## Results

### Overview of the event

**Figure 1:**
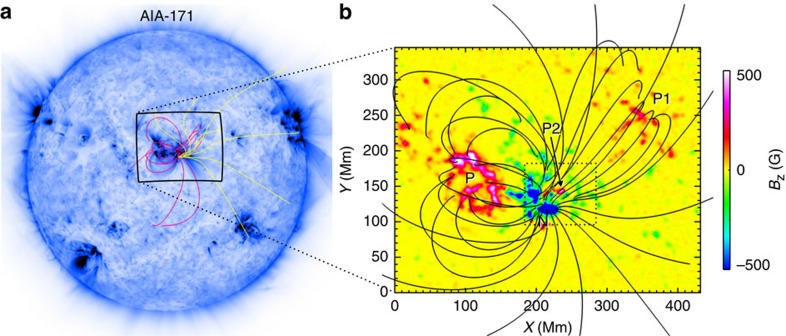
Location of AR 11283 and its basic magnetic topology. (**a**) A full-disk SDO AIA 171 Å image of the Sun observed near the end of 5 September 2011 (day 2). Overlaid on the image are selected magnetic field lines of global potential-field-source-surface model with the pink (yellow) colour denoting closed (open) flux. (**b**) Magnetic environment associated with the flux-emerging region (FER) which is denoted by the dashed box. The magnetic field lines as shown are calculated by the potential field model in a local Cartesian coordinate system. The background image is the map of photospheric magnetic flux with the main polarities labelled as N, P, P1 and P2, where P2 is the newly emerging one and surrounded by negative flux of N. Temporal evolution of photospheric magnetic field in the FER is shown in Fig. 2. The full simulation volume has a slightly larger size of 460(*x*) × 460(*y*) Mm^2^ and a vertical extent of 368(*z*) Mm.

**Figure 2:**
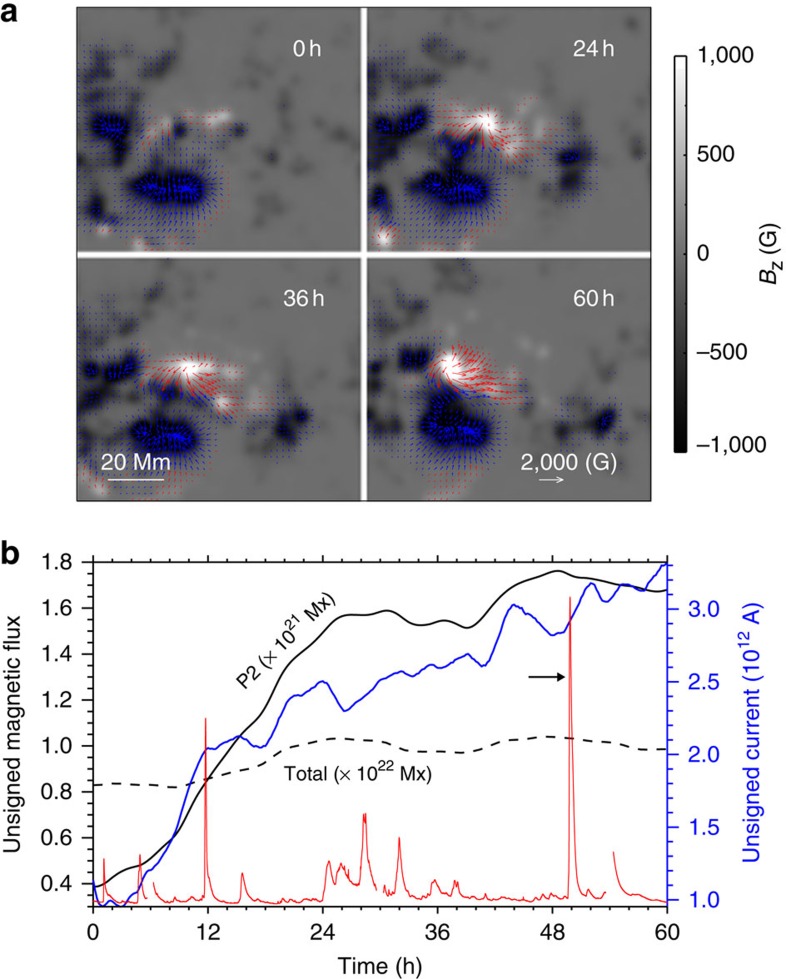
Magnetic field evolution of the FER at the photosphere. (**a**) Evolution of magnetic flux distribution *B*_*z*_ and transverse magnetic components (*B*_*x*_,*B*_*y*_) shown by the arrows, which are coloured as red (blue) for regions of positive (negative) flux. Transverse field less than 100 G is not shown. The field of view (FoV) for the selected region is displayed in Fig. 1b (dashed box). Time starts from 00:00 UT on 4 September 2011. (**b**) Evolution of unsigned magnetic flux for the emerging positive polarity P2 (black solid line) and the whole region shown in **a** (black dashed line). The blue line shows unsigned electric current crossing the photospheric surface of P2, which indicates an increase of the non-potentiality of the coronal magnetic field. The GOES soft X-ray (SXR) flux is also shown as the red line. The arrow denotes the M5.3 flare produced by the FER, while the preceding flares recorded are not related with this region.

NOAA AR 11283 is one of the very flare-productive ARs in solar cycle 24. From 6 to 8 September 2011, four Geostationary-Operational-Environmental-Satellite (GOES) M- and X-class flares occurred successively in this AR, roughly separated by 20 h between one another^33^. Here we follow the evolution of a flux-emerging region (FER, see Fig. 1) in this AR early from 4 September 2011 (day 1) to the onset of its first flare and CME on 6 September (day 3). In this time period, the AR was passing the central meridian of the solar disk as viewed by the Solar Dynamics Observatory (SDO) spacecraft, thus providing an uninterrupted window for measuring the changes of the photospheric magnetic field by the Helioseismic and Magnetic Imager (HMI)^34^ instrument onboard SDO. The basic magnetic configuration of the AR, as shown in Fig. 1, consists of two main polarities, a positive one in the east (P) and a negative one in the west (N). Part of the negative flux also connects to a positive polarity remotely in the northwest (P1). In addition, a global coronal-field extrapolation using the potential-field-source-surface model^35^ indicates the probable presence of open flux (field lines extending beyond the corona to interplanetary space) from N. Starting from day 1, evolution of the photospheric magnetic field is dominated by a parasitic positive polarity (P2) emerging into N (Fig. 2 and Supplementary Movie 1). New flux is injected mainly in day 1, then followed by a fast shearing motion of P2 with respect to N. At the beginning of day 1, magnetic configuration of the FER is close to a potential-field state as the electric current crossing the photospheric surface is very small. Also a non-linear force-free reconstruction shows that its free magnetic energy accounts for a tiny fraction of its total magnetic energy^36^.

**Figure 3:**
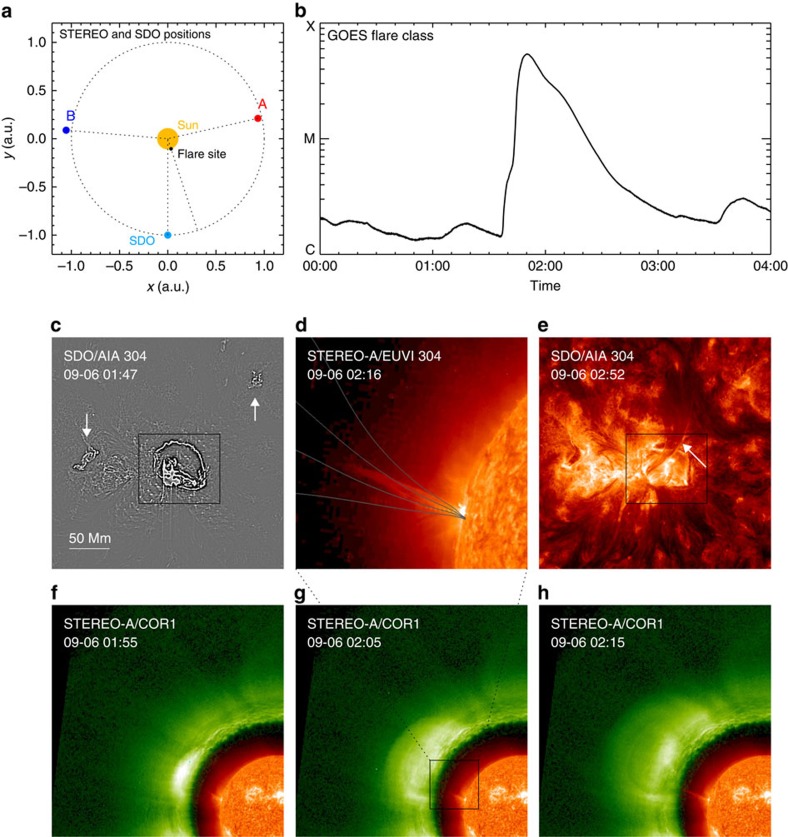
Observation of the flare and filament ejections leading to CME. (**a**) The positions of the Sun, SDO and STEREO-A/B satellites on 6 September 2011. (**b**) The GOES SXR flux around the flare time with the flare class labelled. (**c**) Enhanced image in SDO/AIA 304 Å channel near the peak time of the M5.3 flare. It shows a central circular flare ribbon and two patches of remote flare brightening (marked by arrows). (**d**) STEREO-A extreme ultraviolet imager (EUVI) 304 Å image of the first filament ejection, which starts at the flare peak time and can also be seen by AIA until 02:20 UT. Overlaid are the open magnetic field lines that are also shown in Fig. 1 but now with the same view angle as STEREO-A. (**e**) Of the same FoV in **c**, AIA observation of the second filament ejection (marked by arrow) from the northwest around the circular ribbon, which can be seen from 2:30 UT to 3:00 UT (Supplementary Movie 2). The boxed regions in **c**,**e** denote the same FER shown in Fig. 2a. (**f**–**h**) Combined images of coronagraph (COR1) and EUVI 304 Å observations from STEREO-A showing filament ejection and CME. The boxed region in **g** shows the FoV of **d**.

During the first 2 days there is no eruption from the AR as observed by the Atmospheric Imaging Assembly (AIA) telescope onboard SDO. Early on day 3 a major flare occurs (Fig. 3 and Supplementary Movie 2), which starts at 01:35 UT and peaks at 01:50 UT, reaching a magnitude M5.3 as recorded by GOES. Interestingly, the flare emission consists of a quasi-circular ribbon^37^ enclosing the newly emerged polarity P2 and two small remote brightening patches outside of the circular ribbon, one at polarity P and the other at P1. A slow CME is initiated immediately after the flare peak time from the AR as observed by the Solar Terrestrial Relations Observatory (STEREO) spacecraft in side views of the Sun. Also there are two filament ejections: the first one starts at the flare peak time from the southern corner of the circular ribbon, and the second one starts at 02:30 UT from the north around the circular ribbon. The apparent path of these filament ejections is nearly linear without twist or rotation and it co-aligns well with that of the open flux from N, suggesting that the open flux might be involved with the eruption and provides a channel for the escape of the ejecta. There appears to be secondary EUV brightenings in the declining phase of the main flare corresponding to the small bumps of the GOES X-ray flux (for example, at 02:10 UT), while our study will focus on the main flare event.

### Data-driven simulation

Our simulation starts from the beginning (*t*=0) of day 1, when the FER is almost current-free. The MHD model is initialized with a potential field extrapolation^38^ from the vertical component of the photospheric field (Fig. 1) and a highly tenuous plasma in hydrostatic, isothermal state (with solar gravity) to approximate the coronal low-*β* plasma condition^39^. Then we drive the model continuously by supplying the bottom boundary with data stream of photospheric vector magnetograms from day 1 to 3. The HMI provides routinely high-quality vector magnetograph data at the photosphere with spatial resolution of 1 arcsec and cadence of 12 m, which is adequate for tracking the relatively long-term (hours to days) evolution of AR magnetic structures from formation to eruption. To ensure the input of boundary vector field self-consistently, we utilize the method of projected characteristics which has its foundation on the wave-decomposition principle of the full MHD system^40^. It has been shown that such method can naturally simulate the transport of magnetic energy and helicity to the corona from below^40^,^41^. The unit time in the model is set as *τ*=90 s. By considering that the magnetic evolution at the photosphere is far slower by more than several orders of magnitude than in the corona, we enhance the evolution speed at the bottom boundary of the model by 40 times for the sake of saving the computational time. By this, we assume that 1 h in the HMI data accounts for 1*τ* in the model. More details of the model can be found in the Method section.

### Energies and magnetic helicity evolution

**Figure 4:**
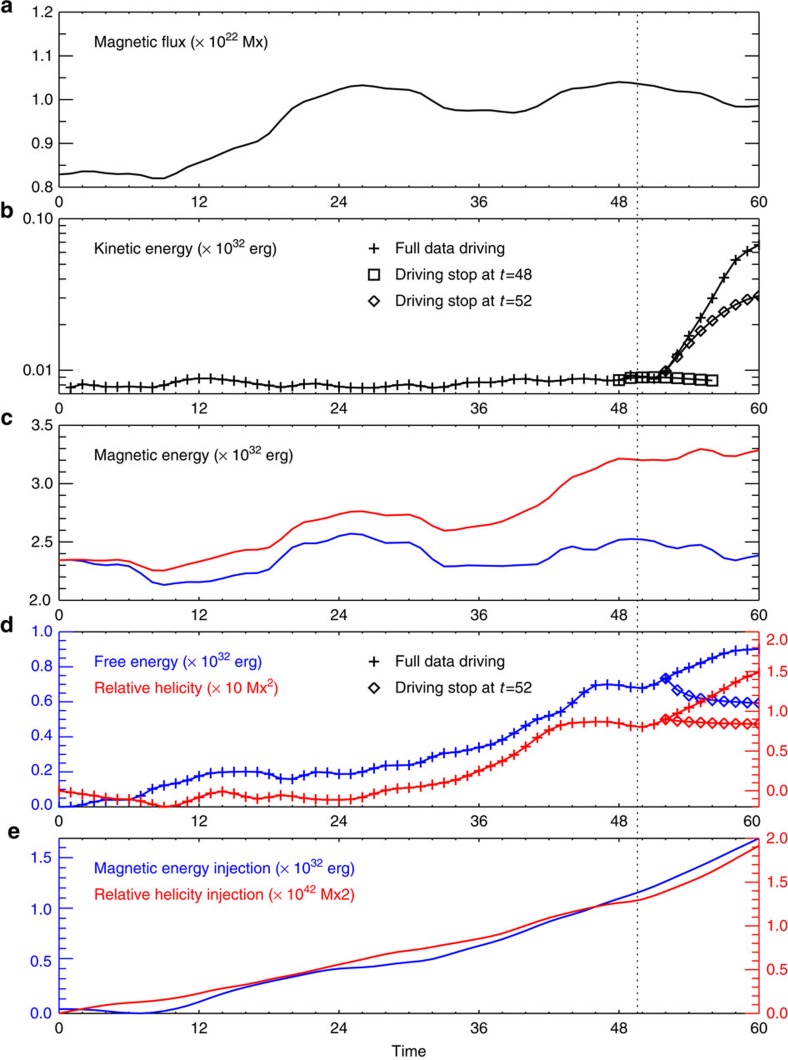
Temporal evolution of energy and related quantities derived from the data-driven MHD simulation. (**a**) Total unsigned magnetic flux. (**b**) Total kinetic energy. In addition to the run with bottom driving supplied all the time, two experiments are carried out: one (the other) with driving ended at *t*=48 (*t*=52), slightly before (after) the eruption onset. (**c**) Magnetic energies derived from the MHD model (red) and potential field model (blue) with the same magnetic flux distribution on the photosphere. (**d**) Free magnetic energy and relative magnetic helicity. (**e**) Estimated amount of injected magnetic energy and helicity through the bottom boundary. All the values are calculated within a sub-volume defined by the FER shown in Fig. 2a with a vertical extent of 100 Mm. The vertical dashed line through the figures denotes the start time of the GOES flare.

When monitoring the temporal evolution from *t*=0 to *t*=60 (in unit of *τ*) for different energies of the MHD system (Fig. 4), we find that its dynamics consist of two distinct phases, a quasi-static evolution phase (from *t*=0 to *t*=51) and an eruption phase (after *t*=51). Furthermore, the onset time *t*_c_=51 of the modelled eruption matches that of the observed flare eruption with a lag of <2*τ*. This suggests that the key transition of dynamics from pre-eruption to eruption is correctly captured by the simulation.

In the first phase from *t*=0 to *t*_c_=51, the coronal MHD system evolves stably in response to the changing of the photospheric field. The kinetic energy keeps a rather low value (compared with the magnetic energy) without noticeable variation. On the other hand, there is continuous injection of magnetic energy through the bottom boundary derived from the Poynting flux. Most of this added energy goes to the non-potential energy (that is, the free energy), especially on the second day, when the fast shearing motion of the emerging polarity commences. During this phase, the free magnetic energy, which can be used to power eruptions, is accumulated to an amount close to 10^32^ erg.

**Figure 5:**
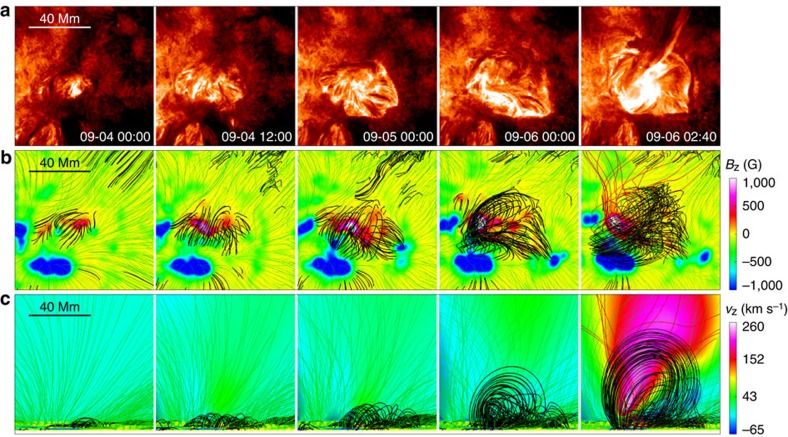
Comparison of the simulated coronal magnetic field of the FER with the SDO/AIA observations. (**a**) AIA 304 Å images at different times from the initial emergence to the eruption. (**b**) Top view of the corresponding magnetic field evolution at different times (*t*=0, 12, 24, 48 and 57) from the MHD model. The field lines are traced from footpoints evenly distributed at the bottom surface, which is shown with the photospheric magnetic flux map. Field lines closed (opening) in the box are coloured black (green), while those becoming open from the closed during the eruption are coloured red. (**c**) Side view of the magnetic field lines from south (that is, the horizontal and vertical axes are *x* and *z*, respectively). The background shows a 2D central cross-section of the 3D volume and its colour indicates the value of vertical component of velocity.

From the time *t*_c_=51, the kinetic energy begins to rapidly rise resembling an exponential growth, and within a short time interval from *t*=51 to 60, it increases by about 1 order of magnitude. This clearly indicates that the system runs into a loss of quasi-equilibrium, that is, a fast eruptive state, which is confirmed by tracking the evolution of magnetic field configuration (Fig. 5, Supplementary Movies 3 and 4). Note that even through the eruption, the magnetic free energy keeps increasing due to the uninterrupted injection of energy into the volume. In addition, we carried out two experimental runs of the model (Fig. 4b), the first (second) with the photosphere driving ended slightly before (after) *t*_c_. In the first run, the kinetic energy decreases eventually without any sign of eruption, while in the second run it evolves similarly as in the case of full-time driving, indicating that the eruption can only occur with the data driving supplied through the critical time point *t*_c_. In the second run, the magnetic free energy drops as expected during the eruption (Fig. 4d). The released magnetic energy is on the order of 10^31^ erg, which is comparable with the energy budget for M-class flares^42^.

Besides the free energy, the relative magnetic helicity is also an important indicator of the non-potentiality of the magnetic field by quantifying the magnetic twist and writhe^43^. Figure 4d shows that the relative helicity evolves in a similar way as the free energy because of the similar injection of helicity flux through the bottom boundary (Fig. 4e). Some observational and theoretical studies^44^,^45^ indicate that there is a threshold (0.25±0.05) for the ratio of relative helicity to the square of total magnetic flux, and eruption seems to occur only when this threshold is exceeded. The estimated value of this ratio near the eruption is about 0.01, which is far below the aforementioned threshold. Such inconsistence might suggest that here the eruption is not directly driven by magnetic twist (or flux rope), and an analysis of the specific topology is required.

### Magnetic topology evolution

**Figure 6:**
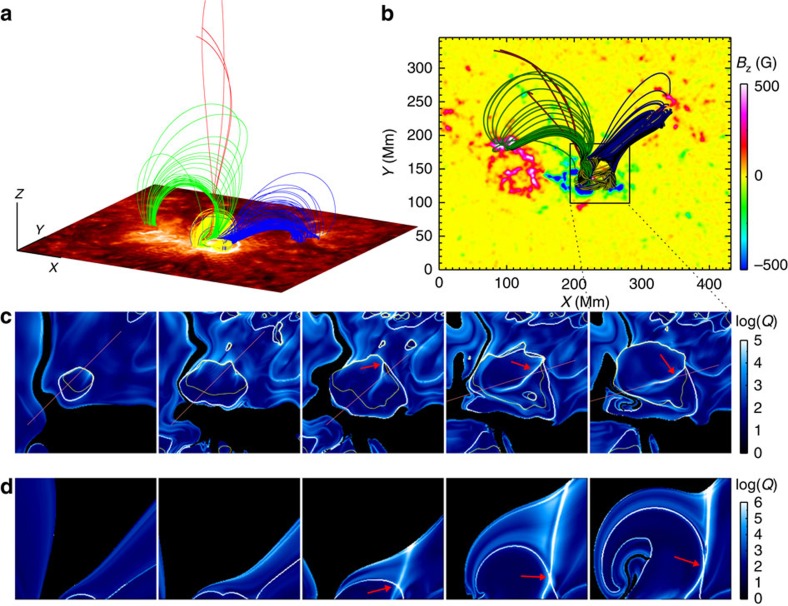
Magnetic topology evolution. (**a**) Sampled field lines traced from topology separatrix at modelling time *t*=57 (with different colours denoting different connectivity as shown). They represent the field lines undergoing reconnection during the flare. At the bottom is AIA 304 Å image near the flare peak time to show the flare ribbons. It can be seen that the locations of flare ribbons are matched well by those footpoints of the reconnecting field lines. (**b**) Top view of the field lines with the background image showing the photospheric magnetic flux. Extents shown in **a**,**b** are identical. (**c**) Magnetic squashing degree log(*Q*) at the bottom surface of the FER (FoV is marked in **b** by the box) at different times (*t*=0, 12, 24, 48 and 57). The separatrix is distinctly revealed by the quasi-circular narrow line with log(*Q*)>5. The black regions represent footpoints of open flux. The yellow lines are PILs. The arrows denote the newly-formed QSL. (**d**) Squashing degree maps for vertical cross-sections whose locations are denoted by the oblique lines shown in **c**. The arrows denote the X-like configuration that is formed along with the new QSL.

In most part of the corona, the plasma is frozen with the magnetic field and so the observed filament-like plasma emission outlines well the geometry of the magnetic field lines. Figure 5 and Supplementary Movie 3 show that overall the simulated magnetic configuration and its evolution resemble the AIA images from emergence to eruption. To characterize the magnetic topology, the squashing degree (*Q*) of the field lines is calculated to locate the important topological structures like separatrices and quasi-separatrix layers (QSLs)^46^,^47^. By this, we find that the emergence of magnetic polarity P2 results in a topological separatrix like a closed dome separating the new emerging flux from the pre-existing one (Fig. 6). As can be seen, the closed field lines with connection to the newly emerging polarity are encircled within the separatrix, while outside of it are pre-existing field lines, and with the increasing of the new flux the separatrix expands in both area and height. Quiescent magnetic reconnection should occur at the separatrix for the successive replacement in the corona of the old flux with the new one^48^. Probably as a result of heating by such reconnection, the separatrix location is manifested in the EUV image (AIA 304 Å) as a bright kernel expanding with time (Fig. 5a). Such a distinct evolving feature is usually observed when new flux is emerging into a region of opposite polarity^49^,^50^.

**Figure 7:**
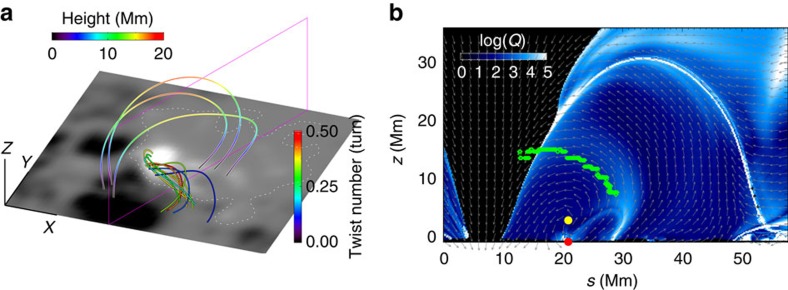
Magnetic field configuration of the emerging sheared structure at the eruption onset time *t*=52. (**a**) Sampled field lines with the low-lying ones as the strongly sheared field, and the overlying ones as its strapping field. Colour of the sheared flux denotes the magnetic twist number of the field lines. Colour of the overlying lines denotes their height. The photospheric magnetogram is shown at the bottom with white dashed line as the PIL. (**b**) A central cross-section of the field whose boundary is denoted by the vertical magenta box in **a**. Its background shows the squashing degree map. The arrows show the direction of magnetic vector components transverse to the cross-section, which form a helical shape centred at the thick yellow dot. Such centre can be regarded as the axis of a magnetic flux rope that may be formed by the twisted field lines. Decay index is computed for a number of paths from the bottom PIL point (the red dot), and a threshold of torus instability is marked by the green diamonds, at which the value of decay index is 1.5.

Another important feature of the emerging field is its growing shear. As can be seen along the south part of photospheric polarity inversion line (PIL) separating P2 and N, the chromospheric filament threads (and the corresponding magnetic field lines) become more and more co-aligned with the PIL. Such stressing of the magnetic field corresponds to the continuous increasing of its free energy and relative helicity (Fig. 4). Here the shearing process does not produce a fully formed magnetic flux rope in the model. Otherwise there should be a distinct QSL wrapping the rope^51^,^52^, which is not seen in the model (Fig. 7). We further estimate the magnetic twist number of the sheared field, which is found to be lower than a half turn. Thus a rope structure is not yet formed.

**Figure 8:**
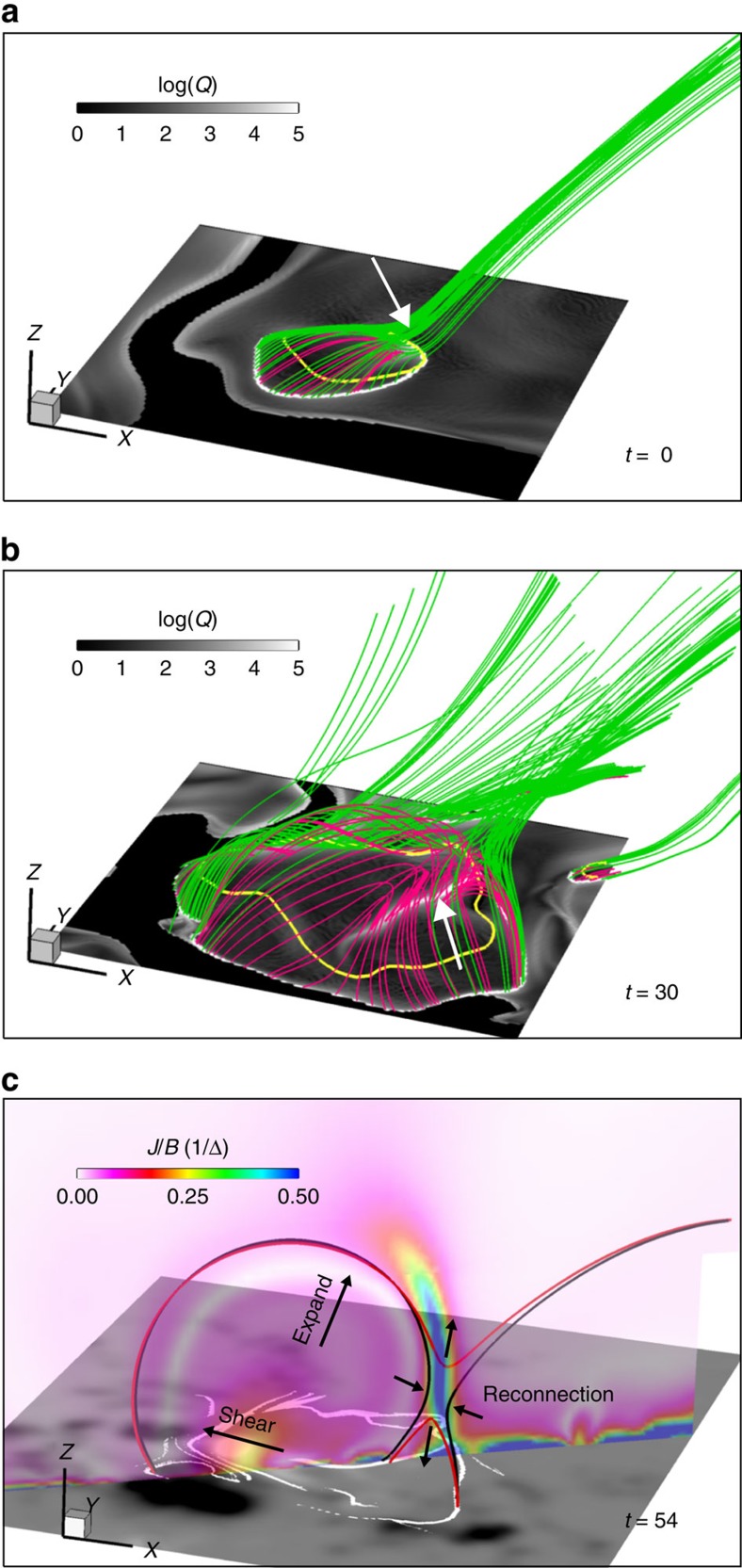
Formation of a jet-like reconnection structure. (**a**) Local magnetic topology at *t*=0 for the newly emerging polarity P2. Squashing degree map is shown on the bottom surface, of which the transverse size is 60 × 60 Mm^2^. Magnetic field lines that form the magnetic topology separatrix surface are traced from the circular line with log(*Q*)>5. The PIL at the bottom is shown by the yellow line. As indicated by the arrow, these field lines become tangential to the bottom surface at the locations where the PIL coincides with the separatrix. (**b**) Same as **a** but for *t*=30, when the new QSL (marked by the arrow) has formed. The closed magnetic field within the separatrix is coloured in red. (**c**) Illustration of the jet-like reconnection as eruption trigger mechanism (simulation time at *t*=54). The transverse size of the bottom surface is 140 × 120 Mm^2^. Field lines in black (red) denote magnetic flux before (after) reconnection. Large arrows denote the bottom shearing and the resulting expansion of the closed arcade. Small arrows indicate the inflow and outflow at the reconnection site. The bottom surface shows the map of *B*_*z*_ overlaid by the white lines showing the trace of separatrix and QSL (log(*Q*)>5). The vertical cross-section false-coloured by value of *J*/*B* shows distinctly a current sheet at the reconnection site. Note that the reconnecting field lines are not coplanar, thus the configuration is fully 3D.

**Figure 9:**
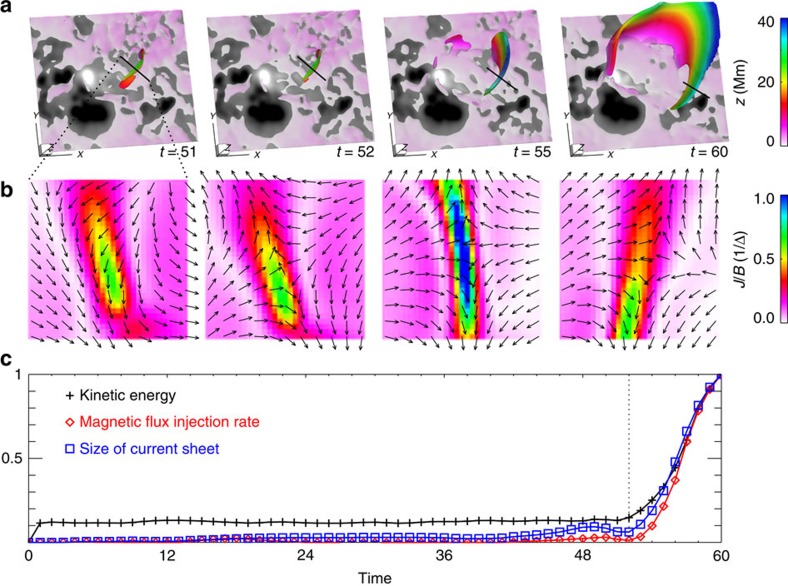
Current sheet development and magnetic reconnection. (**a**) 3D shape of the current sheet. It is defined as the region with *J*/*B*>1/(2Δ) (where Δ is the minimal grid size in the model), which consists of intense current layers with width of ∼Δ, thin enough for resistivity to take effect^70^. Its colour denotes the height *z* from the bottom, and the bottom surface is shown with the photospheric magnetogram of the FER defined in Fig. 2a. (**b**) Flow directions at a vertical cross-section of the current sheet, whose horizontal extent is denoted by the short line in **a**. Reconnection inflow and outflow can be clearly seen after *t*=52. (**c**) Evolution of the size of the current sheet, magnetic flux injection rate (defined by ∮_S_|**B**|**v**d**S** where **v** is plasma velocity and **S** is the full surface of the current sheet) compared with that of the kinetic energy. All are scaled by their values at *t*=60.

With the growing of the newly emerging flux system, part of its edge gets into contact with that of the open flux (Fig. 6c,d, see the changes from *t*=0 to 12). The *Q* maps also show that a new QSL is created during the emerging process. Initially the separatrix surface between the emerging flux and pre-existing one is simply a ‘bald-patch' type^53^, as for the field lines that form the separatrix surface each has one point touching tangentially with the bottom surface. All these points of tangency form a special part of the separatrix at the bottom surface where it coincides with the PIL (see Fig. 8a), and near there its vertical cross-section demonstrates a U shape. After around *t*=24, the new QSL forms, making the topology surface as a mixed type of a bald-patch and an X-line configuration (see Fig. 8b and the Supplementary Movie 5), of which the vertical cross-section appears as an X-shaped structure similar to the topology at a magnetic null point (Fig. 6d). The emergence of such X-line structure provides a favourable configuration for reconnection. The further development of the shearing of the core field increases magnetic pressure and makes its overlying field expand towards the north in the environment of highly asymmetric magnetic flux distribution. This results in a jet-like configuration (as seen in Fig. 8c), in which reconnection can occur between the newly emerged outer arcade (connecting P2 and N) with the side flux of much longer connection paths to polarities P and P1 and even some open flux. Study of electric current distribution in the model shows that a thin layer of intense current (that is, current sheet) is built up at the X-line slightly before the eruption and grows impulsively, extending to almost the whole separatrix surface during the eruption (Fig. 9a). As a result, the global magnetic topology is fully involved in the reconnection (Fig. 6a,b), which provides a plausible explanation of why there forms the circular flare ribbon and additionally the two remote flaring patches. The linear ejection of filaments around the flare ribbon is most likely a result of the opening of the overlying magnetic field, which is reasonably shown by the model. As can be seen in Fig. 5 and Supplementary Movies 3 and 4, the field lines whose colour changes from black to red denote the flux becoming open from closed configuration during the eruption. This is also reflected in the map of squashing degree which shows ‘holes' corresponding to the open flux cutting into the closed circular separatrix.

### Initiation mechanism of eruption

Our modelling results suggest that this eruption is not likely triggered by an unstable flux rope formed prior to the eruption. As mentioned above, the pre-eruptive state is still in sheared-arcade form rather than a well-shaped flux rope. Even if a flux rope exists, it resides far below the critical height for triggering torus instability (Fig. 7), which would occur if the rope axis reaches the height *h* where the decay index (defined by *n*=−*d*log(*B*)/*d*log(*h*)) of the overlying strapping field *B* satisfies *n*>1.5 (ref. 10). The observed features of this eruption are also not consistent with those of flux rope eruptions, for example, the linear shape of the filament ejection does not agree with the eruption of a twisted flux rope, which often demonstrates helical or much more complex structures after being launched^13^,^54^,^55^. We also note that it is the filaments around the circular separatrix rather than along the main PIL (that is, the main body of the possible flux rope) that eject. These filaments are activated possibly due to the opening of their overlying flux, and they may further contribute to the eruption.

Based on the analysis of the magnetic topology from the model, the most appropriate mechanism is that the jet-like reconnection triggers the eruption. This is because once the reconnection sets in, naturally a positive feedback is established between the reconnection, which reduces the inward magnetic tension force that confines the flux below, and the consequent outward expansion of the closed arcades, which in turn enhances the reconnection. Such a mechanism is essentially in correspondence with the breakout eruption model^20^, and here we demonstrate the magnetic configuration in intrinsic three dimensions (3D)^56^. To characterize how fast this reconnection occurs in our simulation, we locate the current sheet (see Fig. 9a) and estimate its size, as well as the rate of magnetic flux injection into the current sheet (that is, the reconnected magnetic flux). It is found that the rate of reconnection is temporally coupled with the acceleration of the plasma (Fig. 9c), clearly indicating the positive feedback between the reconnection and field expansion.

Questions still arise: what makes such reconnection possible and when is it triggered? First there should be a reconnection-favourable topology and this is fulfilled after the X-line magnetic configuration is formed. A further requirement is the building up of a current sheet so that the resistivity is not negligible there and reconnection might happen. By the stressing of the core field which brings field lines of distinctly different directions close to each other along the X-line, such a thin current layer comes into being there at around *t*=46 (see Supplementary Movie 6). To finally trigger the reconnection, the profile of magnetic field across the current sheet needs to be steepened sufficiently (that is, the nearly inversely directed magnetic components on both sides of the current sheet are brought to be close enough to each other) for the numerical diffusion to take effect and ‘merge' the inverse magnetic field components. By analysing the velocity field near the current layer, we find in the model this reconnection is triggered only after *t*_c_=51, because a clear pattern of reconnection inflow/outflow is not seen before *t*_c_ but can be identified shortly afterward. That explains why no eruption occurs when the driving ended at *t*=48, since the reconnection is not yet triggered. This supports that the eruption can only occur after the reconnection (and feedback) is triggered, and once the feedback is established it can eventually cause the eruption even without further surface driving. Here we note that our interpretation for the triggering of the reconnection is restricted within the context of the present numerical MHD model. The other aspects related to the microscopic processes in space plasmas are beyond the scope of the present work.

## Discussion

We have simulated a solar eruption in a realistic and self-consistent way from its origin to onset with a data-driven MHD model. The investigated event consists of a relatively long-duration quasi-equilibrium evolution preceding its eruptive stage of extreme dynamics, and with a single model we are able to calculate the coronal magnetic field evolution for the whole process. The modelled results are supported by the agreement of the magnetic field with EUV images in morphology, the consistency with observation along the timeline from quasi-equilibrium to loss-of-equilibrium, and most importantly, the truly dynamic evolution driven directly by magnetic field data from observation without artificial configuration or constraint.

The modelling offers a reasonable scenario for the eruption. In the background of a multi-polar AR, a small new-flux emergence into the core of the AR leads to the formation of a jet-like configuration that is favourable for reconnection between the newly emerged short arcade and the pre-existing open flux. Meanwhile, the non-potential flux emergence also continuously injects magnetic free energy/helicity into the system due to photospheric shearing motions. Consequently it stresses the field, gradually creating an intense current sheet at the reconnection-favourable site. The system becomes unstable once the reconnection is triggered, since a positive feedback is established between the reconnection and the expansion of the newly emerged arcades. On the other hand, there is no magnetic flux rope fully formed in the modelling, suggesting that a flux rope, although attracting intense interest recently^13^,^14^,^31^, is not a ‘must' for causing a solar eruption. However, ‘on-the-fly' flux rope formation might still happen during the eruption, which again, needs reconnection.

In summary, a data-driven MHD modelling like the one shown here, which is able to realistically simulate the whole process from origin to onset of a solar eruption, can be used as a new way for studying the cause of solar eruptions. Furthermore, utilizing the output of such realistic model as the CME initiation input for models of solar storms travelling from the Sun to Earth^57^,^58^,^59^ will be, we believe, a step forward in developing sophisticated modelling for space weather.

## Methods

### MHD model

We numerically solve the full set of time-dependent, 3D MHD equations with the bottom boundary condition driven continuously by the changing photospheric magnetic field from observations. The model does not include the physics of the thin layer (about several Mm) from the photosphere and chromosphere to the transition region. Otherwise it is required to consider the still unknown mechanism of coronal heating to explain how the temperature increases steeply from thousands of degrees to millions. Even more, the ionization degree at the photosphere is extremely low, making the MHD model inappropriate^60^. Instead, we set the bottom boundary of the model at the coronal base (where the temperature is already at a level of 10^6^ K) and use the magnetic field measured on the photosphere as a reasonable approximation of the field at the coronal base. The plasma thermodynamics is simplified by an adiabatic energy equation as we focus on the structure and evolution of the coronal magnetic field and its interaction with plasma, which dominates the basic dynamics in the corona. No explicit resistivity is included in the magnetic induction equation, and magnetic reconnection is still allowed due to numerical diffusion if the current sheets are thin enough that their thickness is below the grid resolution^9^. A small kinematic viscosity *ν* is used with its value corresponding to the viscous diffusion time (*τ*_*ν*_=*L*^2^/*ν*, where *L* is the unit length) as ∼10^2^ of the Alfvén time (*τ*_A_=*L*/*ν*_A_, where *ν*_A_ is the Alfvén speed) in the strong-field region. The plasma is initialized as in a hydrostatic, isothermal state *T*=10^6^ K (with sound speed *c*_S_=128 km s^−1^) with solar gravity. It is configured to make the plasma *β* as small as 2 × 10^−3^ (the maximal *ν*_A_ is 4 Mm s^−1^) to mimic the coronal low-*β* (highly tenuous) condition^39^. Here the unit length *L* is set as 16 arcsec (or 11.5 Mm), double the length of a basic grid block (8 arcsec) used in the model, and the unit time is set as *τ*=*L*/*c*_S_=90 s.

### Vector magnetogram data

We use the SDO/HMI observation of the photospheric magnetic field^61^. In particular, the Space-weather HMI Active Region Patches (SHARP) vector magnetogram data product ‘hmi.sharp_cea_720 s' (ref. 62) is used to drive the MHD model. With cadence of 12 min and spatial resolution of 1 arcsec, the SHARP data is adequate for simulation of relatively long-term evolution (hours to days) of eruptive AR magnetic structures from their origin to eruption. Furthermore, the SHARP data includes inverted magnetic field data, projected and re-mapped on the cylindrical equal area (CEA) Cartesian coordinate system centred on the tracked AR, which is well-suited for our simulations performed in the Cartesian coordinate system.

Smoothing is needed when data from observation is involved in a computing scheme based on numerical finite difference. Besides, the lower boundary of the MHD model represents the base of the corona rather than the photosphere and the magnetic structures should be broadened from the photosphere to the coronal base. We simulate such broadening using Gauss smoothing of the data with *σ*=2 arcsec as suggested in ref. 63. We also smooth the data in time with Gaussian window of *σ*=4 × 12 min to remove short-term temporal oscillations and spikes due to bad pixels (Supplementary Fig. 1 shows comparison of the data before and after being smoothed).

To fully characterize the related magnetic environment for the eruption, we first cut out a large-scale magnetogram (as shown by the full image in Fig. 1b) from a full-disk HMI data observed near the eruption time (at the beginning of day 3) using the same CEA mapping for the SHARP data. This large map is not changed with time as being a fixed background. Then the sub-area of flux emerging (denoted by the dashed box in Fig. 1b) is replaced by the corresponding SHARP data evolving from day 1 to day 3, and finally the combined maps are smoothed. As can be seen in Fig. 2a and the Supplementary Movie 1, we carefully selected this sub-area to avoid significant flux distributions and changes at its borders. The smoothing further mitigates the mismatch of the evolving embedded sub-area and the fixed background.

### Numerical scheme and boundary conditions

The model equation is solved using an advanced space-time high-accuracy scheme (AMR–CESE–MHD^64^). The computational volume is sufficiently large to enclose the eruptive region of interest and its surrounding magnetic topology of relevance (see Fig. 1), and at the same time consists of a sufficiently small grid size of Δ=360 km (equal to 0.5 arcsec on the Sun) matching that of the HMI pixel. This is realized by a non-uniform mesh based on the magnetic flux distribution. The smallest grid is made around the flux-emerging site, where the photospheric field changes most actively. Grid size is increased gradually to 4 arcsec near the side and top boundaries.

When parallelized with a medium number (for example, a hundred) of CPUs (3 GHz), each time-step advancing of the computing code takes about 5 s. We thus face an extremely time-consuming computational task. On the one hand, our model settings require that the time step (that is, the size of iteration step in time) must be smaller than Δ/max(*ν*_A_)≈0.1 s due to the Courant–Friedrichs–Lewy stability condition^65^. Accordingly, to update in 1 h of real time needs about 50 h of computing time. On the other hand, the self-consistent modelling of eruption initiation requires us to include the preceding long-term energy buildup process for a time scale of days. Thus, the whole evolution process would require months of computing time. To make the computation manageable, we speed up the cadence of inputting the HMI data by 40 times. This is justified by the fact that the photospheric flow speed in accordance with the photospheric field evolution is about 0.1–1 km s^−1^ (refs 66, 67). So in our model settings, the evolution speed of the boundary field, even enhanced by a factor of 40, is still sufficiently small compared with the coronal Alfvén speed (∼Mm s^−1^), and the basic reaction of the coronal field to the bottom changes should not be affected in the non-eruptive time duration. As a result, 1 h in the HMI data accounts for 1*τ* in the simulation. When comparing the simulation with the EUV observations, such scaling also applies to the AIA data in the quasi-static evolution phase, but for the eruptive duration, in principle, time should be scaled according to the ratio of the realistic coronal Alfvén speed to our modelled one. As we have no such data for the real coronal Alfvén speed, we scale the modelling time interval from *t*=49*τ* to 60*τ* as being the real 2 h from 01:00 UT to 03:00 UT of day 3, since this gives a reasonable morphological similarity between simulations and observations from AIA for the eruption process.

The continuous input of boundary vector field to the model is implemented by the projected-characteristics method based on the wave-decomposition principle of the full MHD system^40^. The method can naturally simulate the transferring of magnetic energy and helicity to the corona from below^40^ by self-consistently calculating the surface flow field^41^, which otherwise would have to be derived by local correlation tracking or similar techniques^66^,^68^. Since the cadence of the input data is 12 min, we linearly interpolate the data in time to produce a data set with cadence matching the time step of the MHD model.

### Uncertainty analysis

As being driven directly by data from observations, it is absolutely essential for our modelling that the data are given with good quality and reliability. Here we discuss the possible effects on the modelling results by the known uncertainties and errors of the data.

The SHARP data contains random and systematic errors that may affect our modelling. Estimation of the random errors is included in the data set at each pixel for each magnetic component, that is, s.d. (*σ*). Conservatively, such uncertainty is as much as 200 G in weak-field regions and as little as 70 G where the field is strong (see Supplementary Fig. 2). Accordingly, we test the performance of our modelling with respect to these uncertainties. Due to the limitation of computational resource, we carried out only two experiments but with the data modified to two extremes (or under two extreme conditions): one (the other) with all the magnetic components plus (minus) their s.d., that is, by *σ*, then the modified data are smoothed and input into the MHD model as in the original modelling. Undoubtedly, such kind of modifications to the original data can make systematic changes to the modelling, and moreover the effects accumulate during the long-term run. Supplementary Fig. 3 compares the experiment results with the original one for the kinetic and magnetic free energies, which clearly shows quantitative differences between the results. However, the evolution trend from quasi-static to eruptive states is not changed, and in particular, the critical timing of the eruption onset remains accurate with a small uncertainty of ∼2*τ*. We also compare the magnetic squashing degree maps derived from the experiment results with the original one in Supplementary Fig. 4. It can be expected that the details of the topology will be changed or its shape will be distorted, since, for example, the PIL is modified in the experiments. In particular, the new-emerging area originally enclosed by the PIL expands if we add *σ* to the original data, and it shrinks if subtracting *σ* from the original data. As a result, in the first experiment, it appears that the originally closed separatrix expands and connects to the separatrix in the very weak-field region in the northwest. Nevertheless, the key components constituting the basic topology and their development are still similar to those in the original case. These experiments show that the data uncertainties can quantitatively affect the simulations. However, for the studied event, the main characteristics including the timing of phase transition and the associated dynamic evolution, owing to free energy accumulation and magnetic topology change, remain.

Due to the periodic variation of the SDO orbit, there are daily temporal oscillations of the data that are not removed in the present study. It is estimated^61^ that for the AR strong field (which is of interest in our study), typically such oscillations only cause about ±10∼30 G change (amounts to ±1–2%) of the field strength in a period of 24 h. Such systematic error is even smaller than the estimated random error in the strong-field region. Moreover if compared with the significant change of the new-emerging flux (from nearly zero to the order of 10^21^ Mx) in the 2 days for the specific case here, the change by daily oscillations is sufficiently small. However, the impact can still be seen in the results, for example, the line plots in Fig. 4, as manifested by the small-amplitude undulations on top of the overall gradual changes. In future improvement of the model, we will use the data with the daily oscillations removed as reported recently^69^.

The HMI data might lose its reliability at the flare time due to anomalous flare-related emissions. To examine the robustness of the model with respect to such uncertainties, we assume the flare time (from 1:36 to 2:24 UT on day 3) as a data gap and fill the gap by interpolation in time. Supplementary Fig. 5 compares the simulation results driven by this new data set with those by the original data. As can be seen, the change by this data gap is very limited and does not affect our conclusions. This is because such data gap is very close to the simulated eruption onset, and the eruption-favourable magnetic configuration is already formed.

### Data availability

All the data that are used in current study are publicly available:

The SDO/HMI vector magnetograms and AIA images can be downloaded on the Joint Science Operations Center (JSOC) website

http://jsoc.stanford.edu;

The STEREO/EUVI and COR1 images can be downloaded from

http://stereo-ssc.nascom.nasa.gov/cgi-bin/images;

The GOES X-ray flux data can be downloaded from

http://www.ngdc.noaa.gov/stp/satellite/goes/index.html.

## Additional information

**How to cite this article:** Jiang, C. *et al*. Data-driven magnetohydrodynamic modelling of a flux-emerging active region leading to solar eruption. *Nat. Commun.* 7:11522 doi: 10.1038/ncomms11522 (2016).

## Supplementary Material

Supplementary Information Supplementary Figures 1-5

Supplementary Movie 1 Magnetic vector field evolution of the flux-emerging region observed at the photosphere by SDO/HMI. The transverse components are shown by the arrows, which are colored blue (white) in the positive (negative) flux region.

Supplementary Movie 2 Joint observations of the flare, filament ejections and CME process by SDO and STEREO from different view angles. The GOES X-ray flux is also shown.

Supplementary Movie 3 Comparison of the SDO/AIA 304 Å imaging and the simulated magnetic field evolution for the whole process from the initial flux emergence to the eruption. See Figure 5 for description of the objects as shown for the simulation.

Supplementary Movie 4 Side view of the simulated magnetic field evolution. The background shows a 2D central cross section of the 3D volume and its color indicates the value of vertical component of velocity. The top panel shows the evolution of the kinetic energy scaled by its value at *t* = 60.

Supplementary Movie 5 Rotating view of the 3D magnetic topology at different times. Magnetic squashing-degree map is shown at the bottom and the PIL is shown by the yellow line. Magnetic field lines that form the magnetic topology separatrix surface (and quasi-separatrix layer) are traced from the bottom points with log(*Q*) > 5. The closed magnetic field within the separatrix is colored in red, while outside of it are colored in green. The blue objects are iso-surface of *J/B* = 1/(2Δ), showing the 3D shape of the current sheet. The actual sizes for the three panels (for *t* = 0, 30, and 50, respectively) are identical.

Supplementary Movie 6 Temporal evolution of the 3D current sheet. The bottom surface is shown with the vertical magnetic field component at the photosphere. The color denoted the height from the bottom. See Figure 9 for description of the current sheet.
