# Supplementary material for: Data-driven magnetohydrodynamic modelling of a flux-emerging active region leading to solar eruption
Source: Nat Commun. 2016 May 16;7:11522. doi: 10.1038/ncomms11522 (PMC4873661; doi:10.1038/ncomms11522)
Supplement: Supplementary Information — Supplementary Figures 1-5 [file ncomms11522-s1.pdf]

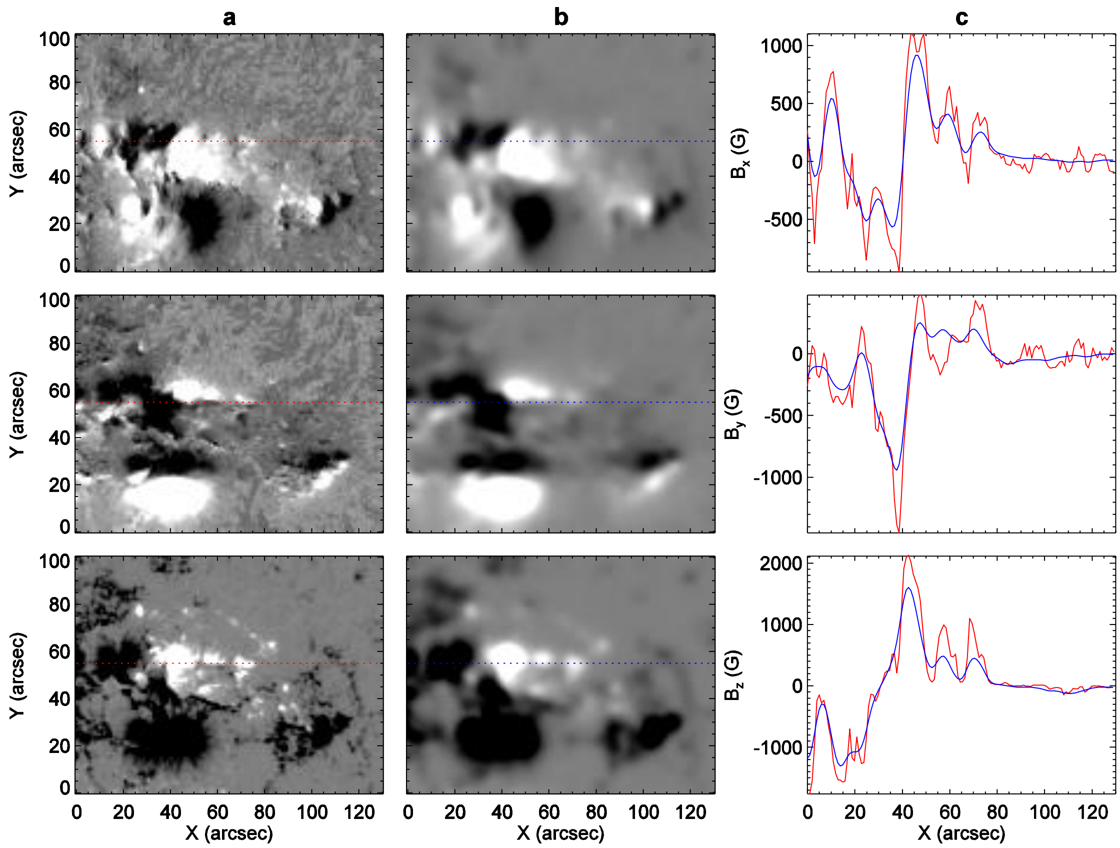

Supplementary Figure 1. Comparison of the original SHARP data before and after being smoothed. The data is sampled at the time of 00:00 UT on September 6, 2011. From top to bottom, panels are shown for the three components of the magnetic field B_x, B_y, and B_z, respectively. (a) The original map. (b) The smoothed map. Colors are saturated at white for value above 500 G and black for value below -500 G. (c) Plots of a random horizontal line, the location of which is shown in (a) and (b). Red (blue) for data before (after) being smoothing.

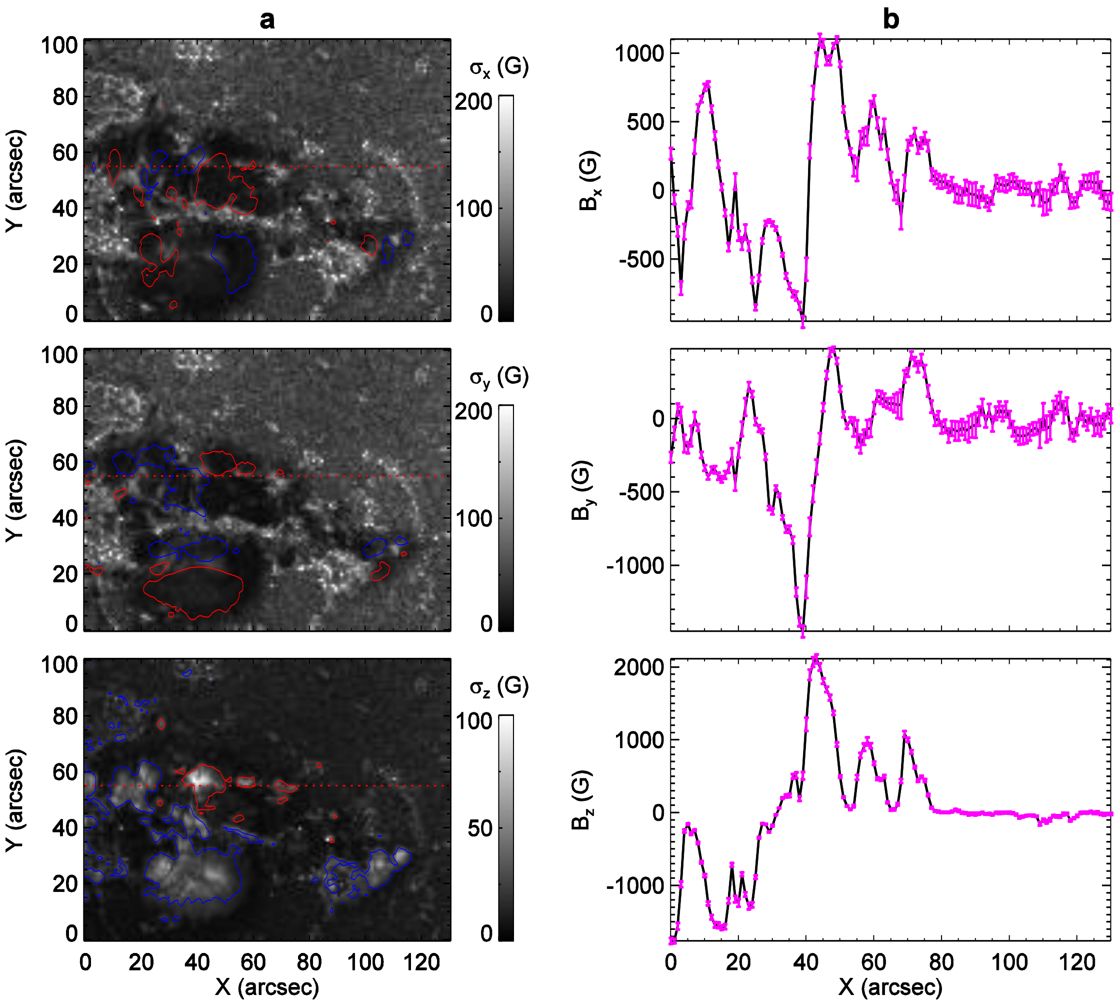

Supplementary Figure 2. Estimated random errors of the SHARP data. The data is sampled at the same time shown Supplementary Figure 1. (a) Map of the errors, i.e., the standard deviations σ_x, σ_y, σ_z, at each pixel for each magnetic component. The contours show the values of magnetic components themselves with red (blue) for 500 G (−500 G). (b) Plots of magnetic field components with error bar on a random horizontal line, the location of which is shown in the (a). From the top to bottom are three components of the field B_x, B_y and B_z, respectively.

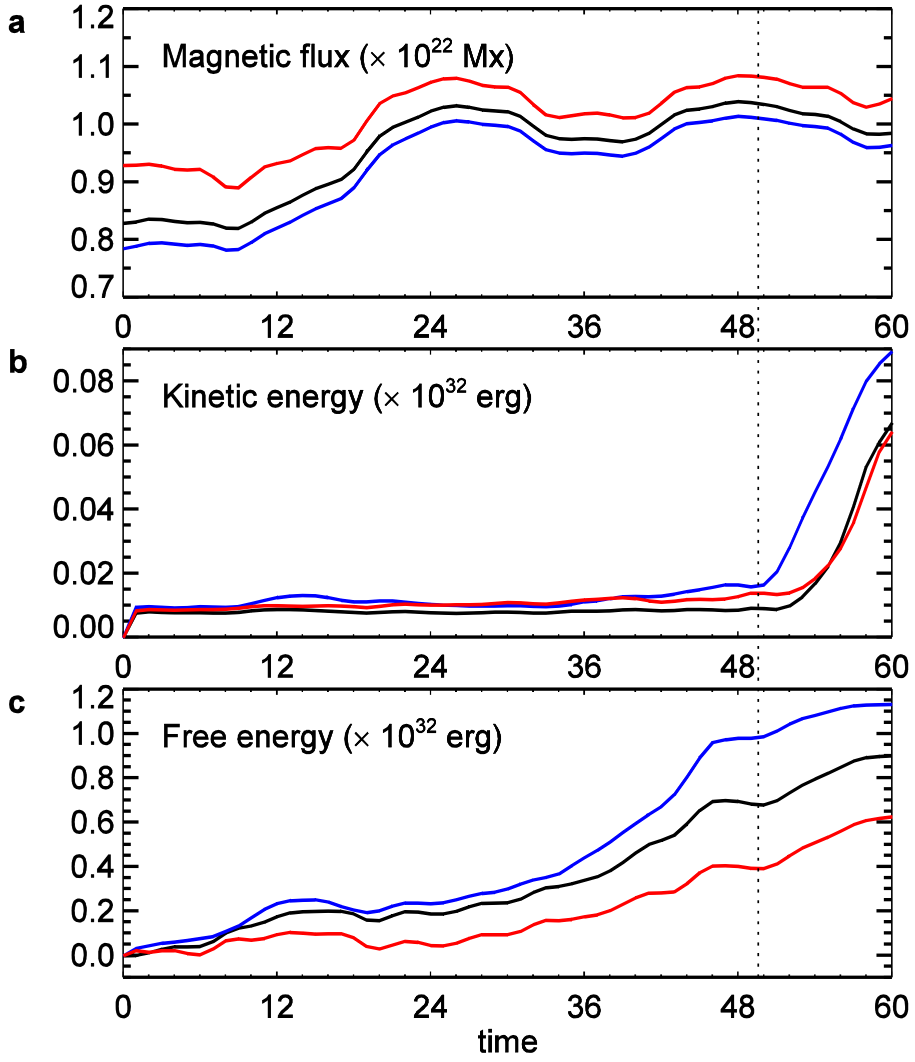

Supplementary Figure 3. Results of two experiments with the data modified extremely: one (the other) with all the magnetic components plus (minus) their standard deviations, shown by the blue (red) lines and compared with the original results (the black line). (a) The unsigned magnetic flux. (b) The kinetic energy. (c) The magnetic free energy.

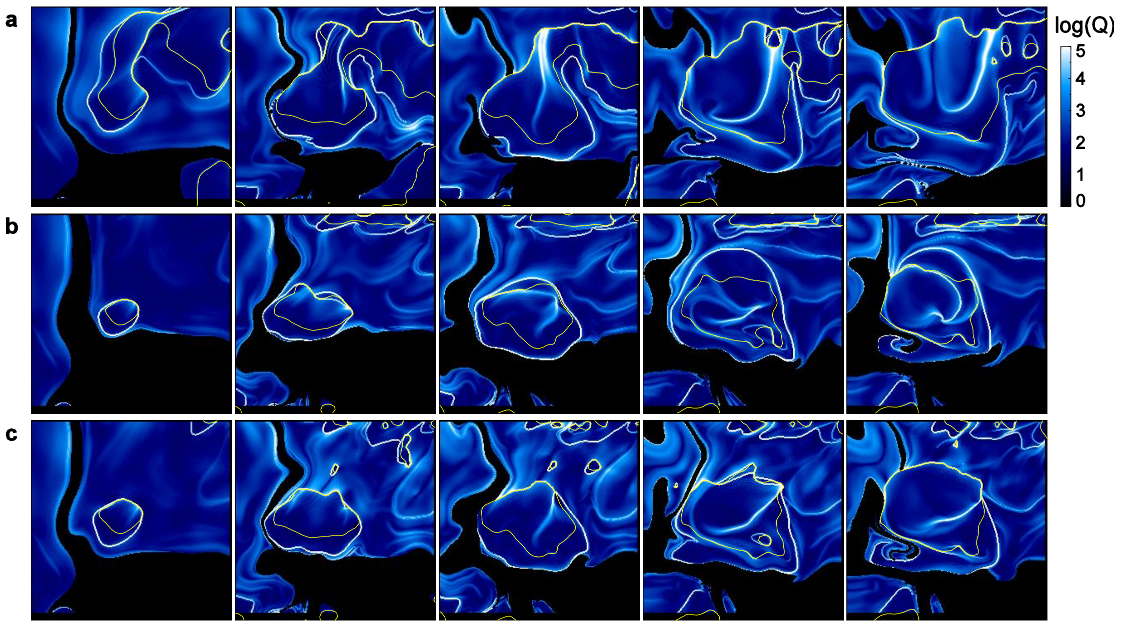

Supplementary Figure 4. Comparison of magnetic squashing-degree maps derived from the experiment results (a and b) with the original one (c). The yellow lines denote the PIL.

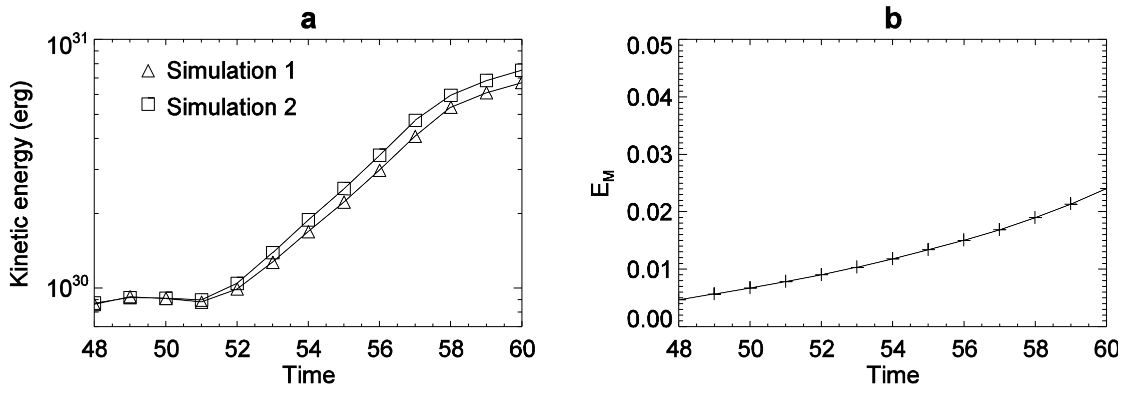

Supplementary Figure 5. Test of the robustness of the model by regarding the flaring time as a data gap of an hour around t = 50. Simulation 1, and 2 are respectively the run with and without data gap**.** (a) Comparison of the kinetic energy evolution. (b) Evolution of the mean relative error for the magnetic field between the results, which is defined as $E_{\mathrm{M}} = \frac{1}{V}\int_V \frac{|\mathbf{B}_1-\mathbf{B}_2|}{|\mathbf{B}_1|}\mathrm{d}V$ where $\mathbf{B}_1$ and $\mathbf{B}_2$ are the magnetic field of simulation 1 and 2, respectively, and V is the computational volume.
